# uPAR-targeted optical near-infrared (NIR) fluorescence imaging and PET for image-guided surgery in head and neck cancer: proof-of-concept in orthotopic xenograft model

**DOI:** 10.18632/oncotarget.14282

**Published:** 2016-12-27

**Authors:** Anders Christensen, Karina Juhl, Morten Persson, Birgitte Wittenborg Charabi, Jann Mortensen, Katalin Kiss, Giedrius Lelkaitis, Niclas Rubek, Christian von Buchwald, Andreas Kjær

**Affiliations:** ^1^ Department of Otolaryngology, Head & Neck Surgery and Audiology, Rigshospitalet, Copenhagen University Hospital, Denmark; ^2^ Department of Clinical Physiology, Nuclear Medicine & PET and Cluster for Molecular Imaging, Rigshospitalet and University of Copenhagen, Denmark; ^3^ Department of Pathology, Rigshospitalet, Copenhagen University Hospital, Denmark

**Keywords:** uPAR, image-guided surgery, tumor margin assessment, head and neck cancer, robotic surgery, PET

## Abstract

**Purpose:**

Urokinase-like Plasminogen Activator Receptor (uPAR) is overexpressed in a variety of carcinoma types, and therefore represents an attractive imaging target. The aim of this study was to assess the feasibility of two uPAR-targeted probes for PET and fluorescence tumor imaging in a human xenograft tongue cancer model.

**Experimental design and results:**

Tumor growth of tongue cancer was monitored by bioluminescence imaging (BLI) and MRI. Either ICG-Glu-Glu-AE105 (fluorescent agent) or ^64^Cu-DOTA-AE105 (PET agent) was injected systemically, and fluorescence imaging or PET/CT imaging was performed. Tissue was collected for micro-fluorescence imaging and histology. A clear fluorescent signal was detected in the primary tumor with a mean *in vivo* tumor-to-background ratio of 2.5. Real-time fluorescence-guided tumor resection was possible, and sub-millimeter tumor deposits could be localized. Histological analysis showed co-localization of the fluorescent signal, uPAR expression and tumor deposits. In addition, the feasibility of uPAR-guided robotic cancer surgery was demonstrated. Also, uPAR-PET imaging showed a clear and localized signal in the tongue tumors.

**Conclusions:**

This study demonstrated the feasibility of combining two uPAR-targeted probes in a preclinical head and neck cancer model. The PET modality provided preoperative non-invasive tumor imaging and the optical modality allowed for real-time fluorescence-guided tumor detection and resection. Clinical translation of this platform seems promising.

## INTRODUCTION

Head and neck squamous cell carcinoma (HNSCC) ranked the 6^th^ most frequent cancer globally in 2013 with more than 800.000 new cases and 360.000 deaths reported annually [1]. In oncological surgery the fundamental principle is complete removal of all cancer tissue to achieve cure. An intended radical procedure has to be balanced against sparing of healthy tissue to maintain an acceptable functional and cosmetic outcome which is especially important in the head and neck region due to anatomical complexity and presence of important neurovascular structures. In most solid cancer types, including HNSCC, positive surgical margins are associated with an increased local recurrence-rate and poor survival outcome and therefore remains a major challenge [2; 3]. The rate of inadequate resection margins, defined as involved or close (<5 mm), in oral squamous cell carcinoma (OSCC) ranged from 30-85% in recent published series [4].

Preoperative imaging modalities (CT, MRI, PET, ultrasound) for staging and planning provide information about tumor location and extension, but current modalities lack sufficient resolution to detect microscopic (subclinical) disease [5; 6]. As a consequence the surgeon intraoperatively still relies on visual inspection and palpation of the tissues to assess tumor borders. Histology of frozen sections is widely used intraoperatively for margin assessment, but the technique is time consuming and is essentially a sampling technique that fails to represent the entire tumor mass and is therefore less accurate compared to postoperative histology workup [7]. Accordingly, there is an unmet need for novel modalities that allows for accurate intraoperative delineation of tumors form healthy tissue to guide tumor resection with clear margins.

Optical near-infrared (NIR) imaging is an emerging technology that enables real-time imaging of injected fluorescent molecular agents accumulated in tissues or structures using dedicated camera systems designed for intraoperative use. Indocyanine Green (ICG) is one of the few clinically approved fluorophores in the NIR spectrum with favorable optical-chemical properties and safety profile [8]. Currently, ICG-based fluorescence imaging has proved clinically feasible for sentinel node biopsy for OSCC and many other malignancies, by our group and by others, where the agent is exploited in a purely non-targeted manner for lymph node detection [9; 10]. The development of fluorescent probes targeted against tumor-specific biomarkers has expanded the research in optical imaging and this may lead to a paradigm shift in oncological surgery. Tumor-targeted fluorescent probes may provide an enhanced contrast between neoplastic and healthy tissue and thereby be an intraoperative tool to guide tumor resection with safe margins and to detect residual disease in the surgical bed [11].

The urokinase-like Plasminogen Activator Receptor (uPAR) system plays a key role in tumor invasion, angiogenesis and metastasis, and high uPAR expression has been reported in many types of carcinoma, including HNSCC [12]. Elevated uPAR activity has consistently been associated with tumor aggressiveness and shown to be a prognostic biomarker for reduced local tumor control and survival outcome, e.g. in OSCC and breast cancer [13; 14]. Importantly, uPAR is located on the cell surface and is regarded tumor-specific because of low or insignificant expression in homeostatic human tissues and is therefore an interesting biomarker for targeted imaging or therapy [15]. The nona-mer peptide AE105 has been described as a ligand with nano-molar affinity for uPAR and has served as a platform in our group and by others, to develop preclinical AE105-based molecular conjugations for tumor-specific PET imaging and therapy [16–20]. A clinical phase-1 study on uPAR-PET using ^64^Cu-DOTA-AE105 in patients with breast, bladder and prostate cancers was recently published by our group with promising results [21]. To explore the translational potential of uPAR-targeted optical imaging we recently reported the applicability of a conjugate of ICG and AE105 (ICG-Glu-Glu-AE105) in a preclinical subcutaneous tumor model [22]. The probe showed high uPAR affinity, plasma stability and favorable optical properties *in vitro*, and *in vivo* tumor-specific binding during real-time fluorescence imaging was demonstrated. Consequently, the purpose of this study was to explore the feasibility of combined uPAR-targeted PET and NIR fluorescence imaging for tumor detection and NIR fluorescence-guided tumor resection in an orthotopic human xenograft model of tongue cancer.

## RESULTS

### *In vitro* uPAR expression of the OSCC cell line

**Figure 1:**
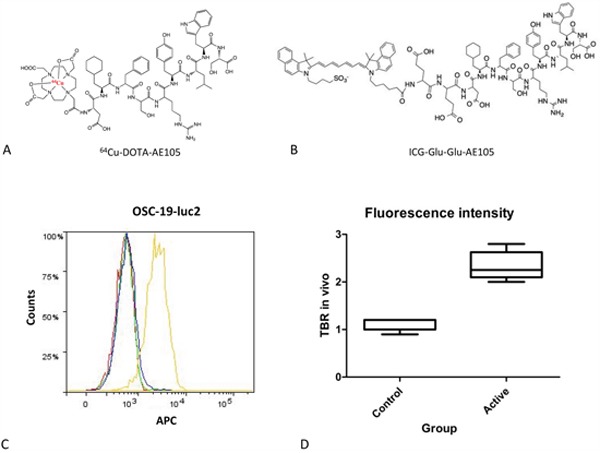
Chemical structure of imaging agents and tumor-target specificity: The chemical structure of ^64^Cu-DOTA-AE105 for uPAR-PET A. and and ICG-Glu-Glu-AE105 for optical imaging B Both agents were designed using the nona-mer peptide AE105 as a scafhold to ensure high binding afffinity to uPAR. Using flowcytometry expression of uPAR on OSC-19-*luc*2 cells was confirmed C. Blank buffer (red), secondary antibody only (green), isotype control antibody + secondary antibody (blue), anti-uPAR antibody + secondary antibody (yellow). Box plot comparing the mean intensity of the fluorescent signal, expressed as TBR, in control animals without tumor and animals bearing a tongue tumor D.

Results from flow cytometry showed a clear right-shift of the fluorescent signal compared to control cells thus demonstrating high level of uPAR expression on OSC-19-*luc2* cells *in vitro* (Figure 1c).

### NIRF imaging and tumor-specificity of ICG-Glu-Glu-AE105

**Figure 2:**
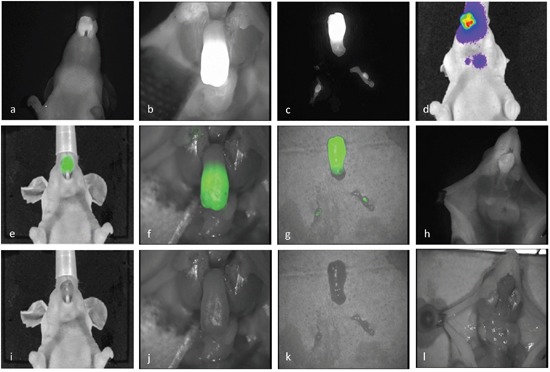
NIRF imaging: Representative imaging captured from one animal Imaging of a tongue tumor *in vivo* **a, e, i**. and *ex vivo* **b, f, j**. The neck was entered ex vivo with a midline incision from the chest to the jaw and the jaw was resected to expose the base and mobile part of the tongue. The tongue specimen and the bilateral lymph node metastases identified and dissected guided by optical guidance **c, g, k**. Preoperatively the presence of tumor and neck metastases was confirmed by BLI **d**. and compared to intraoperative findings with NIRF imaging **h, l**. An example of real-time optical tumor imaging with clinical Fluobeam®800 camera system are demonstrated in supplementary data as a video recording.

In study 1 animals that had OSC-19-*luc*2 cells implanted (n=8) developed a rapidly growing tongue tumor with metastatic spread to first an ipsilateral followed by bilateral neck lymph nodes (n=7) monitored by BLI (Figure 2). Neck metastases were present on day 3 or day 10 in animals inoculated with 10^5^ or 10^4^ tumor cells, respectively. These findings indicate, that the temporal progression of the metastatic process depends on the initial tumor burden in the orthotopic animal model and thus recapitulate the clinical nature of HNSCC. The mean size of the tongue tumors measured on histology sections in study 1 was 4.0 mm (range 3.4-5.2 mm) in the high-dose inoculation group and 2.3 mm (range 1.4-3.5 mm) in the low dose inoculation group and showed involvement of a substantial part of the mobile tongue.

**Figure 3:**
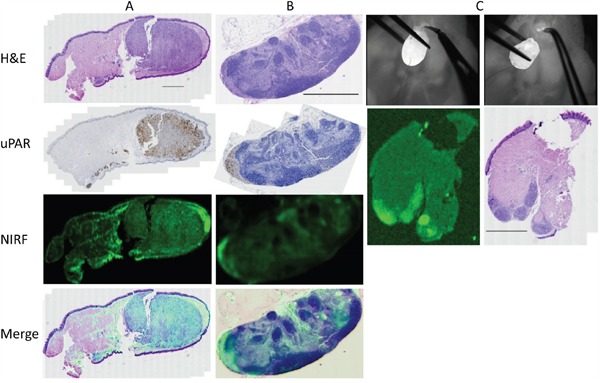
Micro-NIRF imaging and tumor-specificity: Fluorescence imaging of adjacent tissue sections of a large tongue tumor A. and a lymph node with a tumor deposit located in the subcapsular sinus B. subsequently stained for H&E and uPAR In the tongue specimen local spread of tumor nests in the stroma below the mucosa along to entire inferior aspect of the tongue was noted. In the tongue and the lymph node co-localization of the fluorescent signal and the presence of tumor was seen. An example of real-time tumor resection along the demarcation line created by the fluorescent signal where tissue with a clear signal deliberately was left behind in the base of the tongue **C**. On the corresponding histology from the base of tongue residual disease was identified in the inferior part of the tongue which corresponded to the findings on macroscopic and micro-NIRF imaging. The scale bars on the H&E stainings represents 1000 μm.

**Figure 4:**
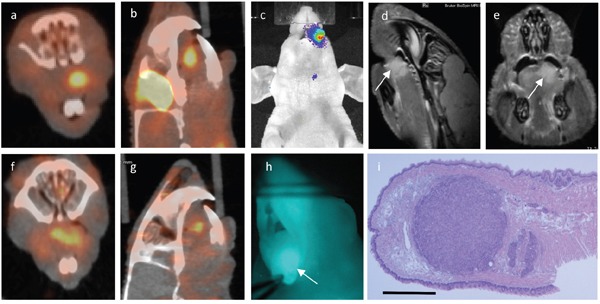
Multimodality imaging: Same animal with a 1.66 millimeter large tumor in the left anterior tongue subjected to ^18^FDG-PET a, b. uPAR-PET f, g. BLI c. MRI d, e. white arrow marking tumor) and uPAR NIRF imaging h. *in vivo* Tissue section stained for H&E **i**. where scale bar represents 1000 μm.

After administration of ICG-Glu-Glu-AE105 optical imaging 12 h post-injection showed a clear and well demarked signal in the tongue of tumor-bearing mice, while no localized signal could be seen in control animals (n=8) *in vivo*. In all animals injected with the optical probe a weak fluorescent signal could be seen universally in the skin and around the nose region. The mean TBR *in vivo* was 2.5 +/− 0.2 using the preclinical IVIS scanner, and 2.3 +/− 0.1 with the clinical camera system, and significantly higher with both systems compared to control animals (p <0.001) (Figure 1D). After the animals were sacrificed and the neck was entered, lymph node metastases were not recognizable while dissecting under bright light illumination, but could only be detected using NIR fluorescence imaging guidance. The lymph node metastases were located in the submandibular region often covered by salivary gland tissue, and the intensity of the fluorescence signal was highly depended on exposure reflecting attenuation of light photons in biological tissue. The intraoperative and *ex vivo* TBRs for detection of tumor and lymph node metastasis were 3.0 +/− 0.3 and 2.2 +/− 0.1 respectively (Figure 2). Histological analysis of adjacent tissue sections stained for H&E and CK confirmed the presence of a poorly differentiated invasive OSCC in the resected tongues and metastatic deposits in the lymph nodes identified by optical imaging. Furthermore, IHC staining for uPAR showed a homogenous expression in the primary tumors and metastasis with staining of the cell membrane primarily. Micro-NIR fluorescence imaging of tissue sections showed co-localization of the fluorescence signal and the presence of tumor compared to non-cancer tissues demonstrating tumor-specific binding of ICG-Glu-Glu-AE105 (Figure 3). In one animal a tongue tumor was intentionally resected exactly along the contrast border created by the fluorescence signal, and a sub-millimeter residual deposit in the tongue base observed by optical imaging was confirmed to be tumor tissue on histology (Figure 4 and Supplementary Video V1).

### Detection threshold of ICG-Glu-Glu-AE105 and MR Imaging for monitoring tumor growth

**Figure 5:**
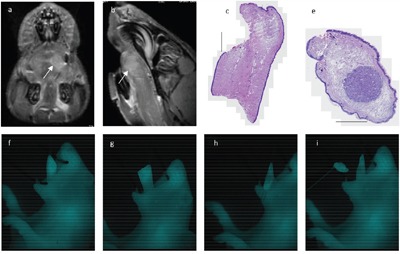
Fluorescence-guided tumor resection: Same animal with a 1.31 mm large tumor in the left anterior tongue as shown on preoperative MRI (a, b, white arrow marking tumor) A time sequence of NIRF imaging where the tongue was fixed with a suture in the tip of the tongue and tumor resection with a small curved scissor was performed guided by real-time optical imaging **f-i**. On histology the tongue specimen showed a clear resection margin, while the resection specimen showed a localized tumor surrounded by healthy tissue showing radicality of the tumor excision.**(c, e)**.

**Figure 6:**
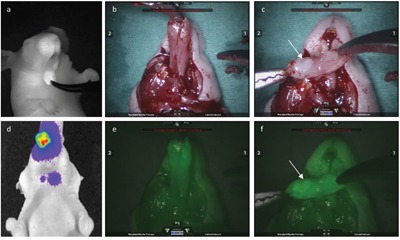
Fluorescence-guided robotic tumor imaging and dissection: Imaging from the same animal with a large tumor in the anterior tongue and bilateral neck metastasis as seen on BLI. d Fluorescence *in vivo* imaging performed with the Fluobeam®800 camera system **a**. and combined fluorescence and color *ex vivo* imaging using the da Vinci®Si™ surgical robot **b, e**. A delineated fluorescence signal from the tumor underlying the mucosa was detected by both NIR camera systems. After transection of the anterior tongue in the midline using the robot a tumor was seen on the resection surface (white arrow) on color imaging **c**. and the tumor margins could be outlined guided by the fluorescence function in the robot **f**. Briefly the da Vinci® Si™ surgical robot has three maneuverable arms for instruments and a maneuverable HD 3D camera, all operated by the surgeon placed in a separate console. Real-time video of the surgical field is presented for the surgeon either as 3D color video or 3D fluorescence imaging. Instant switching between the two modalities is possible to maintain a steady workflow while an accurate dissection is performed.

By using a longitudinal design in study 2 a cohort of animals with a wide range of sizes of primary tongue tumors was established. The maximal diameter of the tumors ranged from 75-2.350 μm measured on histology sections. On MRI the lower threshold for detection was 150 μm, and in animals where serial MRI was performed over time, the individual tumor growth could accurately be visualized (Supplementary Figure S1). Measurement of the tumor size in histology sections and on MRI was highly significantly correlated (p < 0.0001, r = 0.99). On *in vivo* NIRF imaging the smallest detectable tumor was 400 μm in maximal diameter, while tumors below this threshold had a blurred signal and reliable tumor demarcation was not possible. With the assumptions of a mean diameter of the cancer cells of 10 μm, an approximate cell volume of 1 pL and a spherical tumor shape, the cellular detection threshold was 6 × 10^4^ cells. However, when the tongues were transected ex vivo, the intensity of the fluorescence signal increased, and the demarcation line against surrounding normal tissue was sharpened. The mean TBR in study 2 *in vivo* was 1.7 (range 1.3-2.4) in the preclinical IVIS scanner and 1.6 (range 1.1-2.3) with the clinical NIRF camera. As proof of concept, NIRF-guided tumor-resection of small lateralized tongue tumors was performed in a subset of the animals (Figure 5). In two animals additional fluorescence imaging and real-time robotic tumor dissection with the da Vinci robot system was performed. A demarked fluorescence signal in the tongue tumors could be detected and alternate use of the optical and the color imaging functions to visualize tumor while dissecting the tissue was possible (Figure 6 and Supplementary Video V2).

### In vivo uPAR-PET and FDG-PET

After injection of either ^18^F-FDG or ^64^Cu-DOTA-AE105 (N=4) a clear and localized PET signal could be seen in the tongue tumors on PET/CT imaging (Figure 4). The mean TBR measured as SUV max was 2.14 +/− 0.20 for FDG-PET and 2.65 +/− 0.41 for uPAR-PET. The smallest tumor enrolled in the PET protocol had a maximal diameter of 1.66 mm that corresponds to aproximately 2 × 10^6^ cells detected. The tumors could not be deliniated on CT, and were only indentifiable when combined with the PET modality. Lymph node neck metastases could not be detected by any of the two PET tracer agents.

## DISCUSSION

Our results provide the first evidence for feasibility of intraoperative use of an uPAR-directed NIR fluorescent agent imaging for *in vivo* detection and delineation of HNSCC. A sufficient contrast between tumor and healthy tissue could be achieved, and the agent showed evidence of tumor-specific binding on macroscopic and microscopic NIR imaging. The observation, that very small pre-neoangiogenic sized tumor deposits co-localized with the fluorescent signal on micro-NIR imaging was a strong argument for tumor-specificity that makes it very unlikely, that the non-targeted enhanced-permeability and retention (EPR)-effect could be responsible for our findings. In addition, we have provided solid evidence for uPAR-specific interaction of ICG-Glu-Glu-AE105 in a previous publication [22]. As *proof of concept*, the potential synergistic power of combining uPAR targeted non-invasive preoperative PET imaging for planning of surgery and intraoperative optical-guided tumor resection was also demonstrated.

For planning a targeted imaging strategy in a specific type of cancer, essentially the target has to be expressed, and with a high positive expression-rate across a cohort of patients to be of relevance. Target availability within a tumor, expressed as receptor density on the cellular level, is positively correlated with the TBR from an injected imaging agent. Accordingly, our group has previously published preclinical data showing that uPAR-PET can be used quantitatively to measure uPAR tumor expression [19]. In that way uPAR PET can be used as a non-invasive biopsy, and as opposed to a surgical biopsy sample, uPAR PET enables assessment of uPAR expression in the entire tumor volume. Given, that uPAR expression is prognostic for survival outcome in a range of cancers, uPAR PET may potentially be used for risk assessment of cancer patients and to tailor treatment. In relation to uPAR-targeted optical-guided surgery, we believe preoperative uPAR-PET will be of benefit to determine if a tumor is suitable for an optical-guided approach. Both to determine if the tumor is positive for uPAR, and to assess the level of uPAR expression in the entire tumor compartment. It should be noted that with uPAR-PET it is possible to quantify the level of tracer uptake and thereby the receptor density. The relationship between the level of target expression and the magnitude of TBR that can be achieved in biological tissues using injected targeted optical agents is still to be determined. Theoretically, low target expression (receptor density) may result in inadequately low TBR for reliable intraoperative optical margin assessment. However, the detected TBR from an optical agent is influenced by other factors, most importantly the sensitivity of the applied camera system and the clearance of unbound imaging agent in surrounding normal tissue. The fully investigate the impact of tumor receptor density on detected signal intensity for an optical agent will require data from a study in a series of cancer patients. We believe that a uPAR-PET based cut-off value for uptake may be established above which the optical probe will deliver sufficient contrast to guide surgery.

Key factors for successful clinical translation of optical-guided oncologic surgery are the development of agents with a high tumor-binding specificity conjugated to a clinically relevant fluorophore in the NIR spectrum, with sufficient brightness to ensure adequate contrast between diseased and healthy tissue. The activation of the plasminogen system in the process of cancer invasion in numerous types of carcinomas makes uPAR a highly relevant target for therapeutic/theranostic purposes. To our knowledge, this study is the first to investigate uPAR-targeted optical imaging in head and neck cancer. Targeting uPAR with fluorescent probes with various targeting molecules and fluorescent ligands has been explored in a few other preclinical animal studies in mice. Similar to our approach Sun and colleagues recently described the labeling of AE105 with Cy5.5 and optical imaging of subcutaneous flank tumors [23]. Antibodies against uPAR labeled with Alexa Flour 680 or Cy5.5 were used for imaging in prostate, breast and pancreas cancer animal models [24; 25]. Boonstra and coworkers recently published convincing data of optical imaging in colorectal tumor models using an anti-uPAR antibody conjugated to the newly introduced fluorophore ZW800-1 [26]. Another logical strategy has been to label the amino-terminal-fragment (ATF) of uPA, the natural ligand of uPAR. In one study ATF was labeled with a phthalocynine derivate (CPZ) for imaging in a hepatoma model and Yang and colleagues investigated ATF conjugations to Cy5.5 or NIR-830 for optical imaging in animals bearing breast tumors [27; 28]. However, in the above-mentioned studies the fluorophores have never been used in humans, and only ZW800-1 was reported to have passed the first steps for clinical approval. In contrast, ICG is approved for clinical use by FDA and EMA, and the existing clinical data on ICG is substantial, which may offer an advantage in the regulatory process of clinical translation of ICG-Glu-Glu-AE105. Further, all the existing NIR camera systems approved for intraoperative clinical use are optimized for imaging of ICG because non-conjugated ICG already has several clinical applications, e.g. imaging of bile ducts, ureters, renal flow, flap reconstruction and sentinel node biopsy [29].

The development of clinical NIR camera systems that can be readily integrated in a surgical procedure and provide high resolution optical imaging is evenly crucial for the clinical translation of optical-guided surgery [30]. In the present study a clinical camera system designed for open surgery procedures (Fluobeam®800) was used, and to further emphasize the translational potential of the technology, fluorescence-guided surgery using a surgical robot (da Vinci®Si™) was demonstrated. In HNSCC transoral robotic surgery (TORS) is developing rapidly and allows surgeons to resect tumors that previously were technically surgically inaccessible without performing a major surgical invasive procedure [31; 32]. In that context the additional use of cancers-specific optical agents in conjunction with a robotic device to enable accurate image-guided cancer surgery seems very promising and warrants further exploration.

Compared to other imaging modalities, optical imaging may provide detection of very small tumor volumes in the sub-millimeter range, but the clinical detection threshold for cancer tissue for this modality is still to be determined. In a translational perspective, the lower limit for tumor detection for optical imaging agents is important to examine, if the modality is to be used either for detection of residual disease in the tumor bed after resection, or to identify small solitary metastatic deposits in disseminated cancer. It is generally accepted, that a TBR > 2 is required to ensure sufficient contrast for reliable optical imaging [33]. The purpose of study 2 was to explore the minimal size of a tumor deposit needed to enable optical tumor detection with ICG-Glu-Glu-AE105. The smallest tumor detected was 400 μm, and generally the TBRs were lower in study 2 compared to study 1, where larger tumors were studied (mean TBR 1.7 vs. 2.5). Light photons have a limited penetration in biological tissues, and the signal intensity depends on the amount of tracer retained in the tissue, and thereby the size of the tumor deposit. In the orthotopic tongue cancer model we used in the current study, human carcinoma cells were implanted below the mucosa of the tongue, and during growth the tumors remained embedded within the tongue muscle without the development of an epithelial lesion. In this way the model did not fully imitate the clinical situation of human HNSCC originating from the epithelium. The carcinoma is known to be present in the surface throughout the course of tumor growth. In study 2 the majority of the tumor deposits were located 2-3 millimeters below the mucosa, and this may explain the moderate TBRs observed. Because of tissue attenuation the true tumor detection threshold may be lower if surface-involving tumor lesions were studied. Importantly for the further translation of fluorescence-guided surgery in to the clinic, some exposure of the cancer-invaded tissue seems to be necessary, especially if the modality is exploited for detection of small solitary tumor deposits. Importantly, the lymph node metastases in the neck of the animals only could be identified by optical guidance. However, some exposure by surgical dissection of tissue was necessary to detect deeply located metastasis by fluorescence guidance. We anticipate that for clinical use, also some dissection of overlying tissues would be needed to provide adequate penetrance of NIR photons to enable optical guidance towards nodal metastatic deposits in the neck. Accordingly, optical-guided surgery seems suited for easy integration in the neck dissection procedure, which is the standard principle for surgical management of neck metastasis in HNSCC.

In a future clinical scenario of optical-guided tumor resection, for example in the oral cavity, optical assessment of the deep margin may be challenged by sup-optimal visual access with the camera system, bleeding and coagulated tissue after electro-cauterization for hemostasis. Adequate design of the camera system to access different regions and cavities of the body is of paramount importance. We hypothesize, that optical imaging of the tumor bed intraoperatively after completed tumor resection to detect small sub-millimeter tumor deposits in the margin will be of great importance. Also direct optical imaging of the entire resection surface of the tumor specimen in the operation room will be of relevance to detected an involved margin and allow for immediate resection of additional tissue in the tumor bed.

Because HNSCC often is accessible for open resection transorally due to direct visualization of the transformed mucosal line in the upper aerodigestive tract, it is very suitable for an optical imaging approach. In preclinical animal models of head and neck cancer, optical imaging agents targeted against α_v_β_3_ integrin [34], cathepsine B, metalloproteinases [16], PARP1 [35], and EGFR [36] have been investigated. In a recent study, Rosenthal and colleagues published *first-in-man* results on safety of an EFGR-targeted optical probe in 12 HNSCC patients [37]. Apart from expression in many types of carcinomas, the plasminogen activator system seems unique because uPAR not only is expressed by tumor cells, but also by tumor-associated stromal cells and immune cells in the tumor microenvironment. This expression pattern enables simultaneous molecular targeting of both the tumor and the stromal compartment, which may be an advantage for imaging and intervention purposes because of high receptor density in the entire tumor volume [38]. Most HNSCC is characterized by a marked desmoplastic reaction within the tumor and at the invasive border and therefore has a substantial stromal component which can be exploited in a uPAR-targeted theranostic strategy [39].

BLI based on genetically transfected tumor cell lines with the luciferase gene is a highly useful tool in preclinical animal cancer research for tumor detection and monitoring of tumor growth, but the modality is not clinically applicable. For estimation of the exact tumor volume BLI is also less accurate, and in the present study the use of MR imaging for exact temporal monitoring of tumor size was demonstrated. In addition, a high magnetic field strength of 7 Tesla enabled high-resolution imaging of tumor-related anatomy and a very low threshold for tumor detection (150 μm). High resolution MR imaging of internally located tumors in animal models offers novel possibilities in animal research, and is directly comparable to clinical oncology, where MR imaging is increasingly used. In the perspective of molecular imaging the fusion of targeted PET tracers, like uPAR-PET, with MRI may have future important implications due to improved anatomical imaging of the targeted tissue structures as reported recently by our group [40].

In the present study tumor detection could be achieved equally with both FDG- and uPAR-PET. However, the targeting mechanisms for the two PET ligands are very different. FDG-PET is based on imaging of glucose metabolism via the glucose-transporter GLUT1, while uPAR-PET is targeted against uPAR, which is a biomarker specific to cancer and associated with invasion. Even though FDG-PET is extensively used in clinical oncology, there are well known limitations due to enhanced glucose metabolism in different non-malignant tissues, and a limited detection threshold for small cancer deposits. Accordingly, FDG-PET currently has very limited role to play in staging of HNSCC due to insufficient diagnostic performance [41]. In contrast, uPAR-PET may offer highly tumor-specific imaging as recently demonstrated in a *first-in-man* study by our group, which could have several implications in staging, risk-stratification, patient-selection and post-treatment surveillance [21]. By adding optical imaging to a uPAR-targeted strategy we demonstrated bridging of the gap between preoperative uPAR-PET imaging for tumor assessment and planning, and intraoperative optical guidance for tumor detection, delineation and resection, based on ligands with an identical binding domain directed against the same oncotarget. When targeted preoperative and intraoperative imaging is closely linked, the additional information may facilitate chances for effective radical surgery of tumor and metastasis.

In conclusion this study demonstrated the feasibility of real-time fluorescence-guided accurate tumor detection and resection in a preclinical head and neck cancer model using the uPAR-targeted ligand ICG-Glu-Glu-AE105. Further we showed the powerful rational of combing preoperative uPAR-PET and intraoperative uPAR-directed optical guidance to plan and perform surgical tumor intervention. Clinical validation trials of uPAR-targeted optical imaging are warranted.

## MATERIALS AND METHODS

### Animal study design

**Figure 7:**
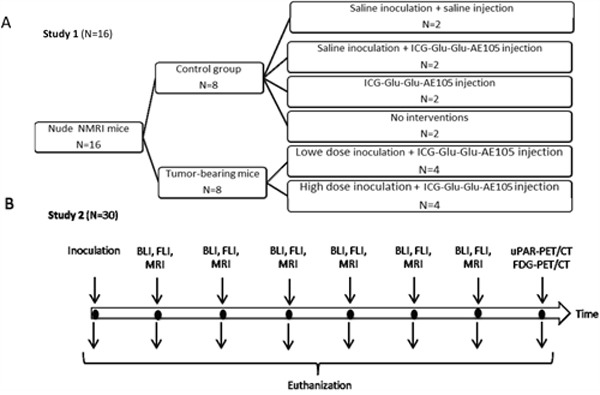
Study design: In study 1 A. animals in the control group and in the high dose inoculation group were sacrificed on day 12 while animals in the low dose inoculation group were sacrificed on day 19 In study 2 **B**. 2-4 animals were sacrificed every 2.-4. day and the last animals were sacrificed on day 17.

Two animal studies were performed to address different research questions (Figure 7). Study 1 was designed to investigate the tumor target specificity, non-specific uptake in healthy tissue and fluorescence intensity of ICG-Glu-Glu-AE105. Study 2 was designed to examine tumor detection threshold of the agent, proof of concept of optical-guided tumor resection, tumor growth monitored by MRI and performance of ^64^Cu-DOTA-AE105 for uPAR-PET in the tongue cancer model. The preparation and characterization of ICG-Glu-Glu-AE105 and ^64^Cu-DOTA-AE105 has been published previously [19; 22]. The chemical structure of the two imaging agents is depicted in Figure 1 (panel A and B).

### Cell line and Orthotopic xenograft animal model

The human OSCC metastatic cell line OSC-19-*luc2* (kindly provided by prof. J.N. Myers, M.D. Anderson Cancer Center, Texas, USA) was cultured in Dulbecco's modified medium (Life Technologies, Paisly, UK), supplemented with 10% fetal bovine serum (FBS), 1% Pen-strep, 1% Sodium Pyruvate and 1% Non-essential amino acid stored at 37°C in a 5% CO_2_ incubator. Female 6 weeks old NMRI nude mice were obtained (Taconic Europe, Denmark) and housed in an experimental animal facility at the University of Copenhagen on a 12 h day/night cycle with food and water available ad libitum. All animal studies were carried out with the approval by the Animal Research Committee of the Danish Ministry of Justice (2014-15-0201-00099). Animals were monitored regularly for weight loss and general health deterioration and euthanized by cervical dislocation. An orthotopic tongue cancer model was established by injection of 10^4^ or 10^5^ OSC-19-*luc2* cells suspended in 15 microliter PBS buffer with a 29 G needle in the left anterior tongue under 4% sevoflurane anaesthesia. Cell Injection was performed either in free hand (study 1) or with a controlled micro pump system (PHD 2000 Infusion/withdraw, Harvard Apparatus Inc., Massachusetts, USA) to achieve minimal hydrostatic injection pressure and accurate tumor implantation (study 2).

### Flow cytometry

Flow cytometry was applied to examine uPAR expression on OSC-19-*luc2* cells. Cells were grown to 70-80% confluence and harvested with 10 nM EDTA and washed in PBS buffer. Cells were stained with Viability Dye (eBioscience, Califonia, USA) and stained with an in-house produced anti-uPAR antibody (Finsen Laboratory, Copenhagen, Denmark), an irrelevant IgG isotype matched control antibody (bs-0295P Bioss, Woburn, MA, USA) or blank buffer control for 30 min in 4°C. Secondary staining was performed with an allophycocyanin (APC) conjugated detection antibody (sc-3846, Santa Cruz, Heidelberg, Germany) for 30 min in 4°C. The result were analysed on a BD FACSCanto™ II (BD Biosciences, California, USA).

### Optical imaging

Bioluminescence imaging (BLI) was applied to monitor tumor growth exploiting the luciferase enzyme inserted in the cell line used. Animals were given an intraperitoneal injection of 150 μL Luciferin (150 mg/mL) (PerkinElmer, Massachusetts, USA), and 10 min post-injection imaging was performed with the IVIS Lumina XR system and for image processing the acquisition software Living Image was used (PerkinElmer, Massachusetts, USA). The BLI signal was quantified by the total photon flux (photons/sec) in regions of interest (ROIs) of the tumor and neck metastasis.

For fluorescence imaging animals were given a tail vein injection of 10 nmol ICG-Glu-Glu-AE105 followed by optical imaging 6-15 h post-injection. The optimal time-window was characterized in a previous study and found to be 6-24 h post-injection [22]. First fluorescence imaging was performed in the preclinical system IVIS Lumina XR with the excitation filter at 745 nm and the emission filter set for ICG. Next animals were transferred to a surgical table for imaging with the clinically approved NIR-camera system Fluobeam®800 (Fluoptics, Grenoble, France). The hand-held camera head was mounted on a surgical arm to allow for hands-free surgical procedures during real-time imaging. This system is customized for ICG imaging working with an 800 nm excitation laser and has a 16x optical zoom and functions for image and video recordings. In vivo imaging was followed by ex vivo imaging, and surgical procedures were performed. In two animals in study 2 additional fluorescence imaging and tumor dissection was performed with a clinically approved surgical robot system using non-human instruments (Da Vinci® Si™, Intuitive Surgical, California, USA). Animals were anaesthetized with 2% isoflurane before and during all in vivo imaging procedures.

In vivo fluorescence intensity was quantified as tumor-to-background-ratio (TBR) calculated as a ROI over the tongue divided by a standard background ROI placed over the chest region. In a similar way an ex vivo (intraoperative) TBR was calculated by drawing a standard background ROI over adjacent muscle tissue. Images from the IVIS Lumina XR and the Fluobeam®800 were processed in Living Image and the ImageJ software (1.46r, NIH, USA) respectively.

### MR and PET imaging

MR imaging was performed on a 7 tesla small animal scanner (Biospec 7.0, Bruker BioSpin, Ettlingen, Germany). A Turbo-Rare high-resolution T2-weighted protocol was applied to generate sagittal and axial imaging of the head region. The slide thickness was 0.3 mm and 11 slides were acquired for each orientation. The echo time was 20 ms and the repetition time was 1200 ms. The field of view was 19 × 16 mm with an image size of 120 x120 mm and the read out spatial resolution was 0.158 mm. The accompanying ParaVision 6.0 software (Bruker Biospin) was used for data analysis.

PET scans were acquired in a 10 min static protocol with a microPET Focus 120 scanner (Siemens Medical Solutions, Erlangen, Germany) 4 hours post i.v. injection of a mean of 8.2 (range 7.5-8.9) MBq ^64^Cu-DOTA-AE105 or 1 hour post i.v. injection of a mean of 9.3 (range 6.9-11.0) MBq ^18^F-FDG during 4% Sevofluran anesthesia. Tracer preparations and PET/CT settings were used as described in a previous paper from our group [19]. All data were analyzed using Inveon Software (Siemens Medical Solutions) and tumor signal was expressed as TBR calculated from measurement of SUV max in ROIs in tumor and adjacent tongue muscle.

### Histology and NIRF tissue imaging

In study 1 resected tongues were bisected and either snap frozen in liquid nitrogen or fixated in alcohol and embedded in paraffin. Next 8 μm frozen tissue sections and 4 μm paraffin-embedded sections were prepared, and micro-NIR fluorescence imaging in the Odyssey Scanner (LI-COR Biosciences, Nebraska, USA) was obtained. In study 2 fixated and paraffin-embedded tissue blocks from tongues were step-sectioned at levels of 50-100 μm to obtain 4 μm sections with the greatest extension of tumor tissue. Adjacent tissue sections were subsequently stained for either hematoxylin and eosin (H&E), cytokeratin (CK; rabbit-anti-cow cytokeratin WAA 1:500, z0622, Dako, Glostrup, Denmark) or uPAR (rabbit-anti-human uPAR, 1:100, Finsen Laboratory, Copenhagen, Denmark). Stained sections were scanned to create digital images, and size of tumor deposits was measured in the accompanying Zenn lite software (Axio Scan.Z1, Carl Zeiss, Jena, Germany). Merging of micro-NIR images and digital images of the same sections was done in the Corel Paintshop Pro X5 software (Corel Corporation, USA).

### Statistical analysis

The SAS software package (SAS Institute Inc., version 6.1, USA) was used. Quantitative data were described as mean +/− SEM or range, and groups were compared using a student t-test. For correlation analysis Pearsons test was applied. A p-value of 0.05 was considered significant.

## SUPPLEMENTARY MATERIALS FIGURES AND VIDEOS






