# Supplementary material for: uPAR-targeted optical near-infrared (NIR) fluorescence imaging and PET for image-guided surgery in head and neck cancer: proof-of-concept in orthotopic xenograft model
Source: Oncotarget. 2016 Dec 27;8(9):15407–19. doi: 10.18632/oncotarget.14282 (PMC5362495; doi:10.18632/oncotarget.14282)
Supplement: Supplementary file 1 [file oncotarget-08-15407-s001.pdf]

uPAR-targeted optical near-infrared (NIR) fluorescence imaging and PET for image-guided surgery in head and neck cancer: proof-of-concept in orthotopic xenograft model

SUPPLEMENTARY FIGURE AND VIDEOS

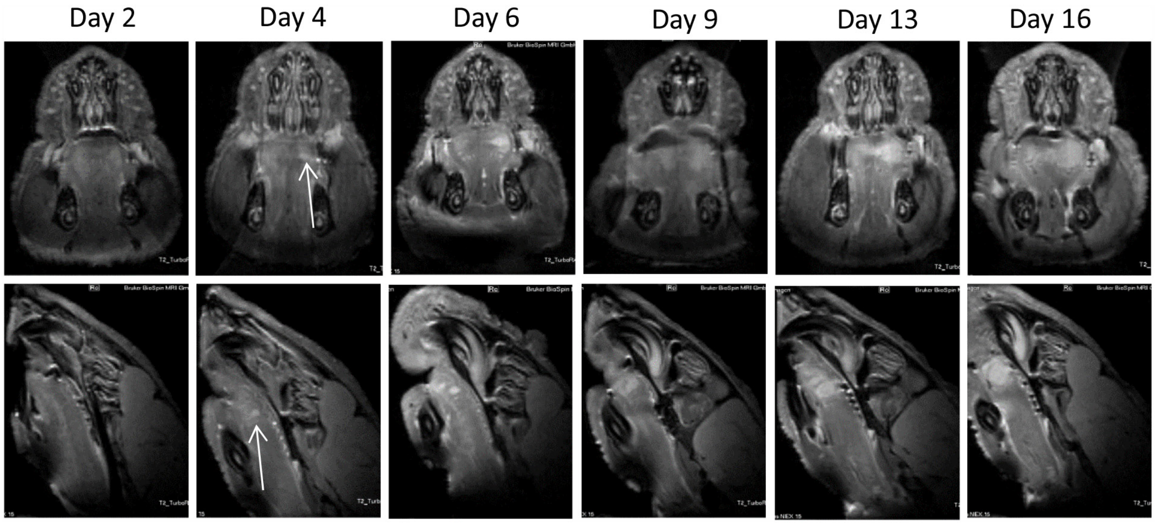

Supplementary Figure 1: MR imaging for monitoring tumor growth: Serial imaging of the same animal in axial and sagittal orientation showing the progressive growth of a tumor in the left anterior tongue. The tumor was detectable from day 4 (white arrow).

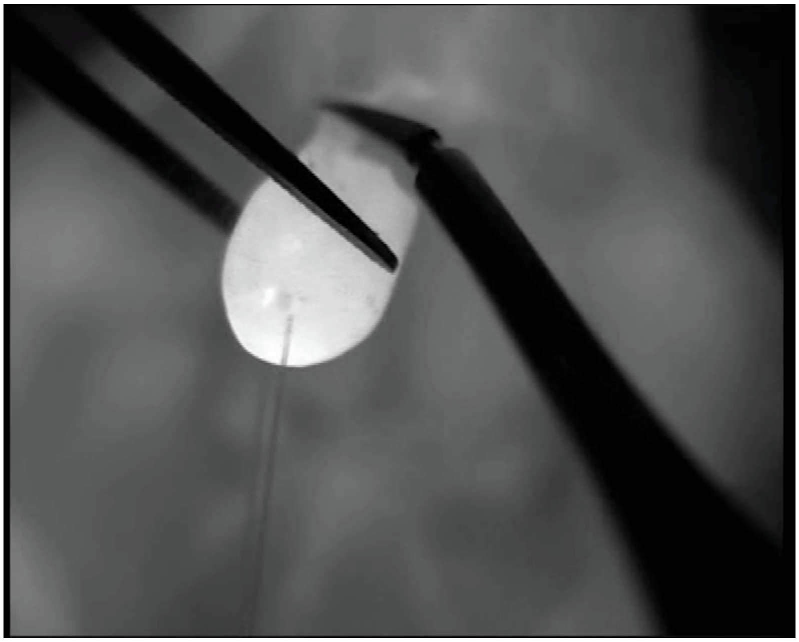

See Supplementary Video 1

Supplementary Video 1: Real-time NIR fluorescence tumor imaging with the Fluobeam®800 camera system: Image-guided resection of a large tongue tumor along the demarcation line created by the fluorescence signal.

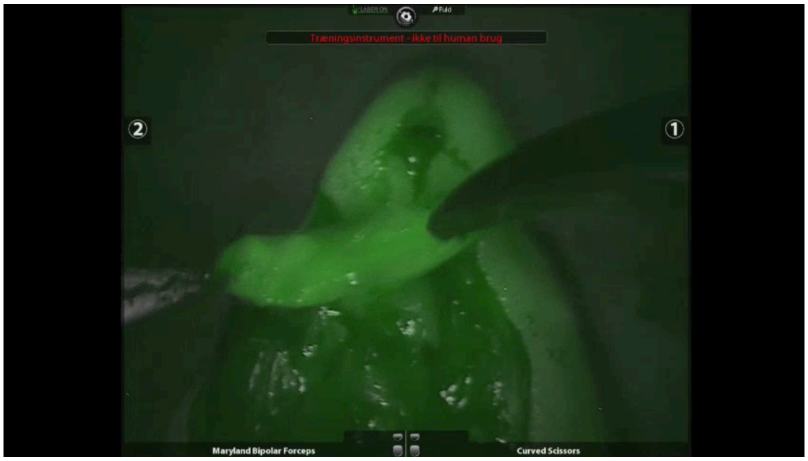

See Supplementary Video 2

Supplementary Video 2: Real-time robotic image-guided tumor imaging: Identification and demarcation of a tongue tumor exploiting the NIR fluorescence imaging mode in the da Vinci robot system.
